# Supplementary material for: Learning curve and surgical outcome of robotic assisted colorectal surgery with ERAS program
Source: Sci Rep. 2022 Nov 29;12:20566. doi: 10.1038/s41598-022-24665-w (PMC9709162; doi:10.1038/s41598-022-24665-w)
Supplement: Supplementary file 2 — Supplementary Information 2. [file 41598_2022_24665_MOESM2_ESM.zip › RAL Raw data-Ñ[▒K/081/OP N_ñw╝╨░O▒Kñσ.pdf]

W52 / W52

臺中榮民總醫院
手術室護理記錄

[redacted]

基本資料

- 室別： 開刀房-17室，手術科別： CRS，手術類別： 常規，日期： 2021/05/08
- 進入時間： 0755 ； 麻醉開始時間： 0805 ； Time Out時間： 0920 ； 劃刀時間： 0920 ； 手術結束時間： 1235 送出時間： 1250 ；
- 麻醉方式： GE
  診斷：CA OF RS COLON
  手術名稱：Robot Assisted Colectomy(L)
  手術局部用藥：NIL
  術前預防性抗生素：
  - 抗生素名稱一： Veterin 1GM IVD 由麻醉護理人員協助執行
  - 時間： 0850
  - 上次簽章: 蘇敬涵

  ，追加預防性抗生素：
- 手術醫師： 蔣鋒帆，楊曙榮，趙英如
- 麻醉醫師： VS 張詒婷
-
  - 刷手護士A ： 黃鈺雯，時間： 0755，刷手護士B ： 何佳青，時間： 0755，接替刷手一： 蘇敬涵，時間： 1200
  - 流動護士A ： 蘇敬涵，時間： 0755，接替流動一： 何佳青，時間： 1130，接替流動二： 蘇敬涵，時間： 1230

皮膚狀況

A-V Shunt： 無
術前： 完整
記錄者： 上次簽章: 蘇敬涵
術後： 完整
記錄者： 上次簽章: 蘇敬涵

手術準備

手術臥式 單一手術臥式
-
  - 手術臥式： 截石臥式
  - 使用附件： 矽膠枕頭，腳架，其它： R17 Lateral support+Silicon Pad*2、R6達文西擺位海棉+墊肩*2
  - 皮膚準備： 10%Aq Betadine，Hibiscrub，2% Chlorhexidine
  - 電燒： 使用電燒: 電極片位置： 右大腿，記錄者： 上次簽章: 蘇敬涵

止血帶T： 無

植入物

- 植入物名稱/號碼/數量/部位：Davinci Xi EndoWrist Stapler 45 Instrument/45-4.3*1/S colon

，記錄者： 上次簽章: 蘇敬涵

標本送檢

- 細菌培養 無
- 細胞學 無
- 冰凍切片 無
- 病理標本
  組織名稱一：IMA LN
  ，
  組織名稱二：IMA root
  ，
  組織名稱三：S colon
  ，更多組織名稱
  -

- 組織名稱四： Distal cut end

- 截肢 無

標本交付 標本： 1 袋 ， 標本： 0 盒 ， 標本： 3 瓶 ， 細胞學： 0 管 ， Culture Tube： 0 管 ， 被交付醫師： R 趙英如 ，

- 記錄者：

傷口術後敷料

傷口敷料： 傷口敷料： 無

- 請於圖上標示部位：

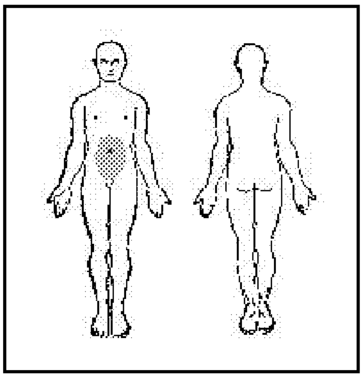

- 傷口敷料： 軟性敷料

導管

導管	天數	導管資訊	插入日期	插入時間	更換日期	拔除日期	拔除時間	拔除原因	預計拔除/更換	預計拔除/更換日期
Epidural PCA(1)	1	部位:硬脊膜外;	2021/05/08	0820						
Foley(1)	1	號碼:16 Fr.; 部位:膀胱;	2021/05/08	0850						
A-line (1)	1	號碼:20 G; 部位:左手;	2021/05/08	0830						
Double Lumen CVC(1)	1	號碼:8 Fr.; 深度:15cm; 部位:jugular (右); 象限:RU;	2021/05/08	0840						

用物計數

- 手術中出血量： Minimal c.c.
- 手術中尿量： 100 c.c.
-
  - 其它： 無

用物計數： 單一切口

- 1.器械： 正確 ，2.縫針： 正確 ，3.刀片 ： 正確 ，4.紗布： 正確 ，5.腹部墊： 未使用 ，6.紗球： 未使用 ，7.PEANUTS ： 未使用 ，8.紗條： 未使用 ，9.絲帶： 未使用 ，10.針頭： 未使用 ，11.Vessel loop： 未使用 ，12.Boots： 未使用 ，13.Jelco： 未使用 ，14.棉條： 未使用 ，
  - 第一次：
    - 刷手護士 ： 黃鈺雯
    - 流動護士： 上次簽章: 蘇敬涵
    - 時間： 1200

  ,
  - 第二次：
    - 刷手護士 ： 黃鈺雯
    - 流動護士： 上次簽章: 蘇敬涵
    - 時間： 1215

  ,
  - 第三次：
    - 刷手護士 ： 黃鈺雯
    - 流動護士： 上次簽章: 蘇敬涵
    - 時間： 1230

備註

備註： R17 Davinci 30度Scope NO.1

- 記錄者： 上次簽章: 蘇敬涵
- 時間： 0944

填寫護理人員 蘇敬涵

正式摘要

W52 / W52

doctorRank:B
